# AECuration: automated event curation for spike sorting

**DOI:** 10.1088/1741-2552/adaa1c

**Published:** 2025-03-24

**Authors:** Xiang Li, Jay W Reddy, Vishal Jain, Mats Forssell, Zabir Ahmed, Maysamreza Chamanzar

**Affiliations:** Department of Electrical and Computer Engineering, Carnegie Mellon University, 5000 Forbes Ave, Pittsburgh, PA 15213, United States of America

**Keywords:** spike sorting, unsupervised learning, signal processing, automatic curation

## Abstract

*Objective*. This paper discusses a novel method for automating the curation of neural spike events detected from neural recordings using spike sorting methods. Spike sorting seeks to identify isolated neural events from extracellular recordings. This is critical for interpretation of electrophysiology recordings in neuroscience studies. Spike sorting analysis is vulnerable to errors because of non-neural events, such as experimental artifacts or electrical interference. To improve the specificity of spike sorting results, a manual postprocessing curation is typically used to examine the detected events and identify neural spikes based on their specific features. However, this manual curation process is subjective, prone to human errors and not scalable, especially for large datasets. *Approach*. To address these challenges, we introduce AECuration, a novel automatic curation method based on an autoencoder model trained on features of simulated extracellular spike waveforms. Using reconstruction error as a performance metric, our method classifies neural and non-neural events in experimental electrophysiology datasets. *Main results*. This paper demonstrates that AECuration can classify neural events with 97.46% accuracy on synthetic datasets. Moreover, our method can improve the sensitivity of different spike sorting pipelines on datasets with ground-truth recordings by up to 20%. The ratio of clustered units with low interspike interval violation rates is improved from 55.3% to 85.5% as demonstrated using our in-house experimental dataset. *Significance*. AEcuration is a time-domain evaluation method that automates the analysis of extracellular recordings based on learned time-domain features. Once trained on a synthetic dataset, this method can be applied to real extracellular datasets without the need for re-training. This highlights the generalizability of AECuration. It can be readily integrated with existing spike sorting pipelines as a preprocessing filtering or a postprocessing curation step to improve the overall accuracy and efficiency.

## 1. Introduction

Understanding the dynamics of neural circuits in the brain is crucial to advance fundamental neuroscience research as well as for the diagnosis and treatment of neurological disorders. Analyzing neural activity through extracellular recordings is one of the most common and powerful tools in neuroscience for this purpose [1]. Extracellular recordings are usually carried out using electrodes implanted into the brain tissue to detect the electrical activity of nearby neurons surrounding the electrodes [2]. These recordings are composed of electrical activity originating from nearby neurons as well as various sources of unrelated signals, including muscle activity and respiration artifacts, electrical interference (powerline interference or interference from nearby equipment), and amplifier noise [3, 4]. A key step in analyzing extracellular recordings is spike sorting. To identify single-unit activity, which is the fundamental component of extracellular recording signals, individual neural action potentials (spikes) are extracted from electrophysiology recordings, grouping them into event clusters called units that represent the activity of individual neurons [5–9].

Since the first microelectrode device was used for extracellular recording [10], the number of simultaneous recording channels has dramatically increased [11, 12]. Electrophysiology recording devices with hundreds of electrodes are now widely available [13–15]. There is a pressing need to implement fully automated spike sorting pipelines to handle large datasets collected from hundreds of simultaneous recording channels. Spike sorting consists of several steps, most of which have already been automated through decades of research [16–18]. Although different implementations of automatic spike sorting algorithms are available, the validation step of curating the analyzed data is still manual [19–21]. A human expert assesses clustered units using visual inspection of waveform shapes and firing patterns. Neural spike waveforms are typically characterized by three recognizable phases: a modest positive peak, followed by a pronounced negative peak, ending with a refractory period [22]. However, manual curation can vary significantly among experimenters, introducing subjectivity and uncertainty [23, 24]. Furthermore, this manual curation process is time-consuming and cannot scale with the ever-increasing size of electrophysiological recording datasets. As a result, manual curation remains the bottleneck of a fully automatic spike sorting pipeline [25].

Recently, several automatic curation methods have been introduced to address this issue [26–28]. Most of these automatic curation techniques focus on the statistical properties of the signal to curate units based on quality metrics [20]. These quality metrics are based on biophysical knowledge of neural firing. A common quality metric is an interspike interval (ISI) violation rate. The lower bound for ISI is dictated by the neuron refractory period [29]. Therefore, the number of violations can be used to evaluate the quality of identified units. Some other statistical analyses include inspecting the cross-correlation [30] and the chi-square test [23, 26]. These approaches exploit biophysical knowledge of neurons and use statistical analysis across many neural spikes to perform unit curation. However, such statistical analysis requires a large sample of spikes, which restricts the usage of these approaches.

Some other curation algorithms focus on the clustering quality rather than the biophysical properties of neurons. Cluster metrics include the variability of residuals and isolation between clusters [31]. With the assumption that the event-to-event variability is only from background noise, the unimodality test can be used to check if the unit contains more than one neuron [32]. Another measurement is cluster isolation distance. Units with a large cluster isolation distance are more likely to represent individual neurons [33]. Several distance measurements have been used to represent cluster isolation distance, such as Mahalanobis distance [34], Fisher’s linear discriminant [20], nearest-neighbors [17], and silhouette score [35]. These metrics primarily focus on the abstract properties of clustering rather than the underlying cell biology, making it hard to interpret the results from a biological perspective.

Ensemble sorting, instead of individual algorithmic approaches, has also been proposed for automatic curation. A fundamental requirement of a good sorting algorithm is stability, which is the consistency of sorting results on the same dataset [36]. By running the same algorithm with various perturbations several times, the stability of units across sortings can determine the sorting quality. Similarly, high-quality units should be consistent across various sorting algorithms and can be identified by simultaneously running several different sorters and aggregating the sorting results based on waveform similarity or spike train agreement [37, 38]. However, ensemble sorting is computationally intensive and requires a much longer time than conventional methods.

Deep-learning-based curation algorithms have also been proposed. The general form of these algorithms is based on training a model on the output of a spike sorting algorithm to learn the behavior of the sorter. The models can then predict whether the input unit is from a single neuron or background noise. The prediction can be based on the raw waveform [39, 40] or the feature space [41]. Supervised learning methods, while effective, require datasets to have labels for every recording. However, in many cases, a well-labeled dataset is either unavailable or insufficient to train a reliable supervised model. In such cases, an unsupervised learning approach can be used to cluster neural spikes and non-neural events without the need for labeled data [42, 43]. Another approach is transfer learning, which utilizes synthetic datasets to train the model and later transfers to real-world applications, which avoids the limited availability of ground-truth datasets [44, 45].

Here, we present an automated data curation method with several important capabilities using an autoencoder. First, it is unsupervised since well-annotated datasets and ground-truth information are not usually available in most real-world scenarios. Unlike previous unsupervised methods, this method does not assume prior knowledge about the recording. Any extracellular recording can be processed without assuming the presence of spikes. Second, the algorithms can evaluate unit quality even in small datasets, where there are only a few recorded spikes, without solely relying on the statistical properties of large groups of spikes. Furthermore, the classification decision based on time-domain patterns is inspired by unit classification approaches of expert electrophysiologists. These electrophysiologists usually classify neural units by identifying time-domain patterns, such as spike waveforms with specific peak amplitudes, rather than relying on abstract statistical properties.

In this study, we use an autoencoder to perform automatic curation. As a generic unsupervised feature extraction method, an autoencoder aims to identify some low-dimensional features of the input data and reconstruct the input based on these extracted features [46]. Such models are widely used in anomaly detection for identifying unusual events in time-series data [47, 48]. The motivation for using such a model is that the learned low-dimensional features are constrained to represent the key regularities of the input data to minimize reconstruction errors. In the case of unusual events, the reconstruction errors will be greater as the autoencoder cannot encode such deviations [49]. Autoencoders have previously been used in other stages of spike sorting, including dimensionality reduction [50] and data compression [51]. Still, to the best of our knowledge, our paper presents the first application of autoencoders to spike curation via anomaly detection.

**Figure 1.**
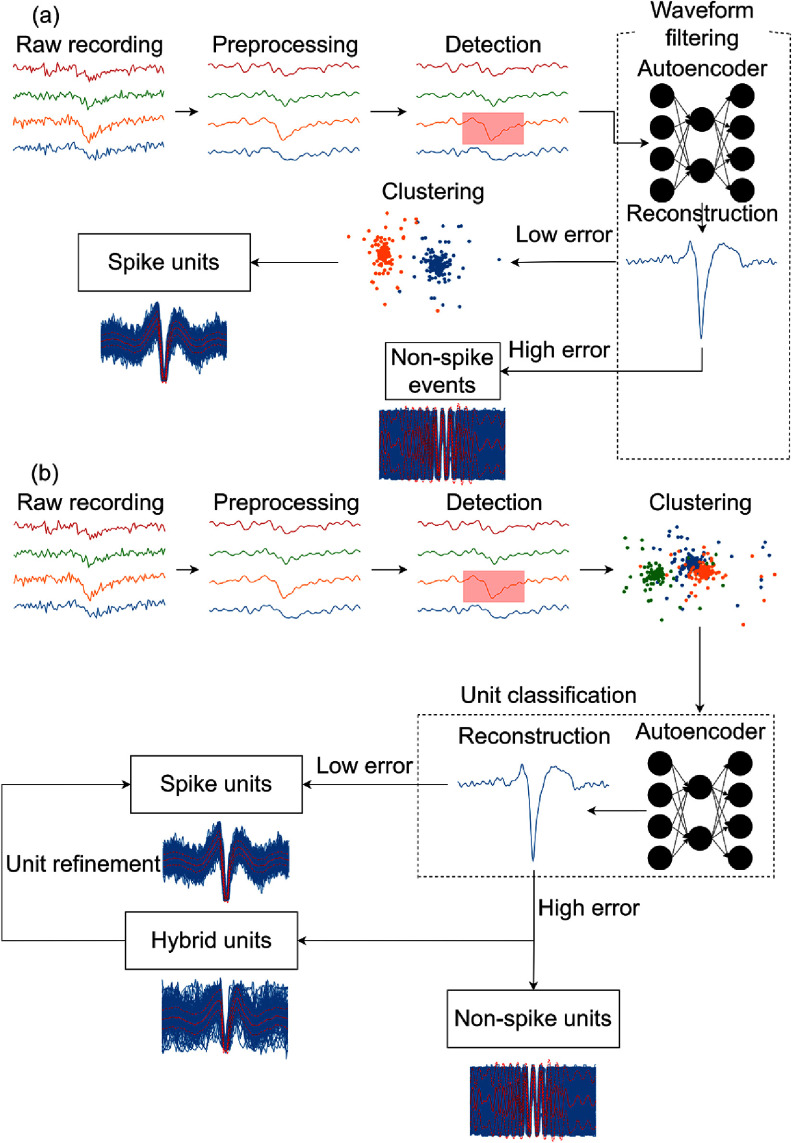
(a) AECuration, applied before clustering, can remove non-neural events from the recording. The time segments detected via threshold-crossings are taken as input to the AECuration model. Only time segments classified as neural spikes are considered in clustering. After clustering, neural units are more likely to be detected since the recordings are filtered to remove noise events. (b) AECuration can refine previously clustered units by classifying and selectively removing non-neural events.

In this paper, we demonstrate a fully automatic curation algorithm based on an autoencoder (AECuration) and evaluate its performance on different experimental electrophysiology datasets. The paper is organized as follows: section 2 describes the design of AECuration, including the autoencoder architecture and how it is integrated into existing spike sorting pipelines as both preprocessing and postprocessing steps (figure 1). Section 3 presents the evaluation of AECuration on both synthetic and experimental datasets. We demonstrate that AECuration can improve spike sorting accuracy across multiple spike sorting algorithms and datasets as both preprocessing and postprocessing. Section 4 discusses the impact of AECuration on improving current spike sorting pipelines and identifies directions for further development.

## 2. Methods

### 2.1. Autoencoder design

#### 2.1.1. Autoencoder model

In the spike sorting pipelines used in this study, each potential spike event is detected based on an amplitude threshold from preprocessed time-series electrophysiological data. These spike events are separated into 3 ms segments of data, including 1.5 ms before and 1.5 ms after each peak. The 3 ms segments are aligned with the peak and each segment is processed in a random order, assuming these segments are statistically independent. The input, denoted as feature vector *z*, is generated through a series of preprocessing steps, including bandpass filtering, common median referencing, and normalization procedures. We utilize an autoencoder to determine whether the original segment corresponds to a neural spike based on a reconstruction error of *z*.

**Figure 2.**
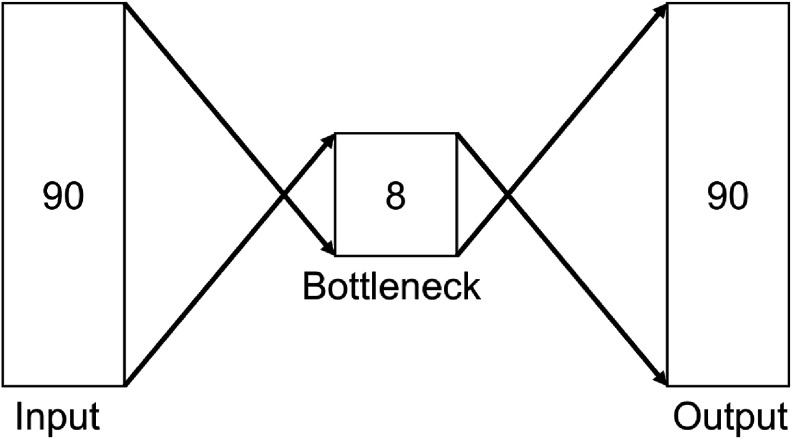
Architecture of the used autoencoder model. The dimension of the input and output layer is the same as the dimension of *z*, which is the sampling rate times the length of the segment. The bottleneck layer has a much smaller dimension and forces the model to encode input into a low-dimensional representation.

An autoencoder is a type of neural network that learns the intrinsic properties of the dataset to efficiently encode input data. The model is forced to compress the input to a low-dimensional latent representation and reconstruct the input based on this representation. Such encoding is optimized by minimizing the reconstruction error between the input and its output. We choose to use an autoencoder, as it can learn to reproduce data that is similar to training data but poorly reconstructs anomalous data that significantly deviates from the training data. The autoencoder in our implementation consists of an input layer, an output layer, and a fully-connected bottleneck layer. The structure of the model is described in figure 2. The model consists of an input and output layer with 90 dimensions, corresponding to 3 ms segments sampled at 30 kHz, along with an 8-dimensional bottleneck layer. The autoencoder is designed to be simple, and yet effective. Increasing the complexity of the autoencoder model can result in overfitting [52]. The autoencoder is trained on a ground-truth dataset, in which all training segments correspond to real neural spikes. The mean squared logarithmic error (MSLE),

\begin{equation*} S\left(\hat{x}, x\right) = \frac{1}{N} \sum_{i = 1}^{N}\left(\log\left(|\hat{x}\left[i\right]| + 1\right) - \log\left(|x\left[i\right]| + 1\right)\right)^2\end{equation*}

measures the difference between the input and the reconstructed output. The score computes the mean of the logarithmic transformation of deviation between the original data *x* and reconstructed data $\hat{x}$. The absolute value and the additional one ensure that there is no undefined negative or zero logarithm. MSLE is chosen because of its logarithmic transformation, which makes this method robust to amplitude fluctuations. This is particularly useful for electrophysiology recordings because the amplitude may vary depending on the experimental configuration [53].

After sufficient training, the model establishes a norm for neural spikes, and the reconstruction error for segments corresponding to neural spikes should be small. Then, we can pass *z* from real-world data through the trained autoencoder and compute the reconstruction error. If the error exceeds a threshold, we can classify *z* and the corresponding 3 ms segment as a non-neural event.

#### 2.1.2. Spike classification

AECuration can quantitatively determine the quality of each detected event based on MSLE (1), which measures the similarity between the original input and the reconstructed waveform. An MSLE threshold can separate the neural and non-neural events, which is useful both for preprocessing and postprocessing and will be discussed in detail later. Here, we introduce our definition of neural and non-neural events.

Non-neural events are classified into two types [31, 54]. Non-biological events (Type I) come from artifacts and interference generated by non-biological sources, such as movement artifacts and electromagnetic interference. This kind of event behaves differently from neural events in the waveform shape. Noisy detected events (Type II) are neural signals highly distorted by noise. Due to their lower SNR, they cannot be considered neural spikes. We consider an event with an SNR lower than 10 dB as a non-neural event. Here, SNR is measured as the spike peak amplitude divided by the standard deviation of background noise when no spike occurs. We choose a high threshold for SNR since spikes with very large SNR are likely to come from a single cell, and we want to preferentially select signals from a single neuron [55]. Spike classification aims to identify neural spikes from non-neural events. In the following analyses, non-neural events refer to both Type I and Type II non-neural events.

#### 2.1.3. Training data (MEArec training dataset)

**Figure 3.**
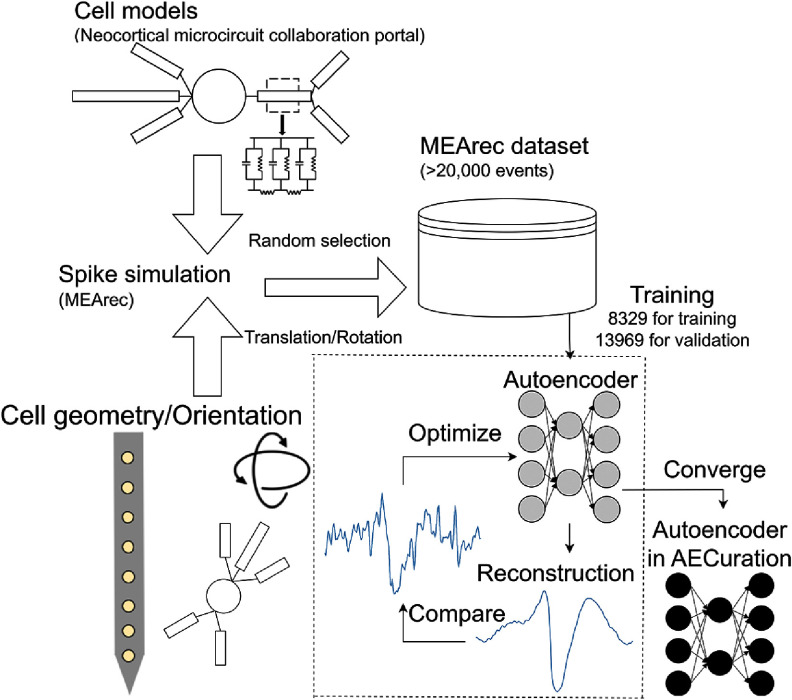
The model is trained on a custom synthetic dataset generated by the biophysical extracellular simulator MEArec [56]. Cell models are chosen from 23 cell model datasets and randomly placed in an 80 *µ*m cube. MEArec generates spike trains according to the cell model and location. The randomly selected cell models with 8329 waveforms are simulated for training and 13 969 waveforms are simulated for validation. Each waveform has a 3 ms time window with one aligned spike.

We trained our autoencoder on a synthetic recording dataset generated by the MEArec simulator, which simulates realistic and customizable electrophysiology recordings (figure 3) [56]. By training the model on synthetic data, it can easily be provided with a large and diverse training set without the experimental limitations to collect such a ground-truth training set. The simulations are generated based on juvenile rat somatosensory cortex cell models from the neocortical microcircuit collaboration portal [57].

**Figure 4.**
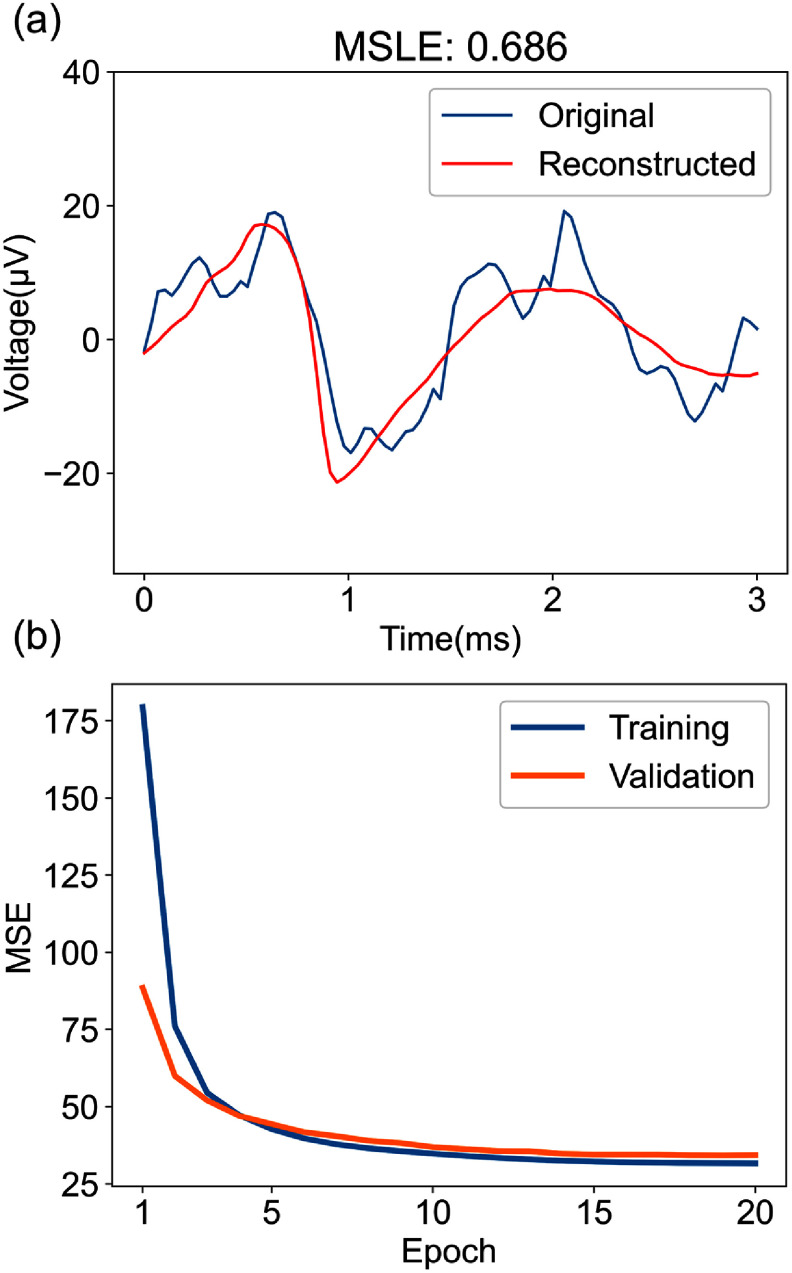
Training of the autoencoder on the synthetic dataset. (a) The autoencoder can reconstruct the input waveform (red trace) by encoding the most relevant regularities in neural spikes. These waveforms feature neural spikes of various magnitudes from the MEARec simulator with added Gaussian noise [56]. The MSLE value reflects the signal variance from the autoencoder’s estimated templates. (b) The training loss and validation loss in mean square error (MSE). The convergence of the loss shows that the autoencoder learns to represent the features of neural spikes and the model is not overfitted.

We synthesized two recordings with additive white Gaussian noise characterized by a standard deviation of 10 *µ*V. The synthesized recordings are based on different probe geometries. One employs a four-channel tetrode configuration with 30 *µ*m of inter-channel distance, while the other uses a $10\times10$ grid square microelectrode array (MEA) configuration with 15 *µ*m of inter-channel distance. The recordings contain 36 and 50 neurons with random cell types, respectively. Cells are randomly placed as rigid bodies in an 80 *µ*m cube centered on the centroid of the probe with six degrees of freedom. Two spike trains of 60 s duration are generated from a Poisson distribution for each recording, with 8329 and 13 969 spikes each, respectively. The recording with 8329 spikes is used for training, and the other with 13 969 spikes is used for validation. the model loss is measured via MLSE and the model is trained until the training loss and validation loss are converged after 20 epochs, as shown in figure 4.

#### 2.1.4. Model convergence

The AECuration is trained and analyzed on the Google Colaboratory cloud platform using an Intel Xeon 2.30 GHz CPU with NVIDIA T4 GPU. The model is built using Tensorflow [58].

The input and reconstructed signals are one-dimensional vectors with 90 samples, representing a 3 ms spike segment sampled at 30 kHz. Successful training of the autoencoder is shown by the convergence of the reconstruction error for both training and validation datasets drawn from the simulation (figure 4).

After training an autoencoder model to reconstruct neural spikes, the method can be integrated into the existing spike sorting frameworks to perform automatic filtering and spike curation. To apply the AECuration to neural recordings, we first need to perform spike sorting. AECuration is compatible with majority of popular spike sorting pipelines, and we use multiple different spike sorting pipelines to demonstrate its generalization. AECuration can be used to identify spike and non-neural events, which makes it suitable for both preprocessing and postprocessing. In the next section, we discuss how it can be used as the input of the neural signal processing pipeline to improve the preprocessing steps.

### 2.2. AECuration for spike sorting

#### 2.2.1. Preprocessing step

**Figure 5.**
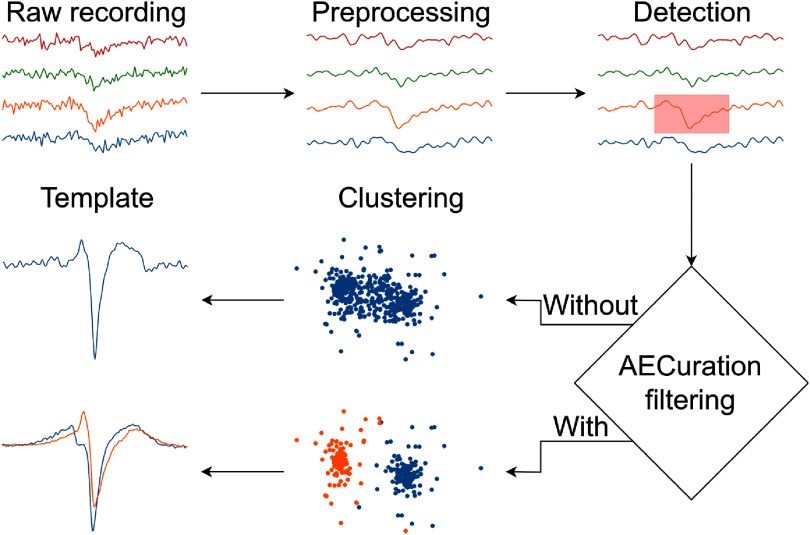
Schematic diagram of preprocessing using AECuration. After event detection and alignment, each event is evaluated by the autoencoder-based filter to determine whether it is a neural spike. Removing non-neural events before clustering can improve efficiency and accuracy since fewer data points are processed, and clustering has less interference.

AECuration introduces an autoencoder-based filter that can be added after the event detection step and before the spike clustering step of a spike sorting pipeline as shown in figure 5. By filtering non-neural events before clustering, AECuration can reduce noise and interference by removing non-neural events before they reach the clustering step. Detected events with MSLE above a threshold are classified as non-neural events and excluded from clustering. Only the remaining events with low MSLE proceed to the clustering step, ensuring that the clustering focuses on high-quality neural spikes (figure 5).

Since AECuration can remove non-neural events, it could work in different stages of the spike sorting pipeline. Preprocessing with AECuration has two major benefits: it can improve efficiency by reducing the number of events to be processed, which makes the clustering faster and more manageable. It can also increase accuracy by reducing noise and interference in clustering, leading to more accurate grouping of neural spikes. In the next section, we discuss the effect of AECuration when used as a postprocessing step after clustering, which is a different application of AECuration.

#### 2.2.2. Postprocessing step

Alternatively, AECuration can be used as a postprocessing step after clustering, which can improve the quality of spike sorting by refining the clustered units. After clustering, not all clustered units are free of non-neural events. It is necessary to review and refine clustered units, a time-consuming manual process. A trained expert inspects the spike waveforms to identify features such as polarity and spike width [59]. As explained earlier, this manual process can suffer from subjective biases and is difficult to scale. AECuration can automatically identify and remove non-neural events, making the post-clustering curation rigorous, repeatable, and efficient. Events are classified based on their MSLE and units are categorized into three types depending on the neural spikes to non-neural events ratio, which can be customized according to the application.

(i) Neural units: contain mostly neural spikes. We set the threshold such that $95 \%$ of the events are neural spikes in our experiment.
(ii) Hybrid units: contain a mixture of neural spikes and non-neural events. We set the threshold such that between $50 \%$ and $95 \%$ of events are neural spikes in our experiment.
(iii) Non-neural units: contain mostly non-neural events. We set the threshold such that less than $50 \%$ of event are neural spikes in our experiment.

The thresholds between different types can be defined based on the distribution characteristics of MSLE. The MSLE of spike and non-neural units follow distinct distributions. In contrast, the MSLE of hybrid units has overlapping characteristics. Once units are classified, different strategies can be applied to retain useful information while discarding interference. Neural units are kept intact, while non-neural units are entirely discarded. Hybrid units, which are challenging in manual curation, will undergo further refinement at the event level to extract neural spikes and discard non-neural events.

Refinement based on AECuration can discriminate neural spikes from non-neural events for individual events within each unit, which is another level of automatic fine filtering. Since units can contain a combination of neural spikes and non-neural events, we can refine the units by selectively removing events with a large MSLE, which is defined as an MSLE larger than 1.5 by default if not specified otherwise. Such events are more likely to be considered non-neural events. After discarding non-neural events, a clean subset consisting of neural spikes is ready for further analysis. This process, which we call unit refinement, ensures that useful neural spikes are extracted from hybrid units and utilized for further research. The combination of unit classification and refinement allows researchers to extract valuable information from units that are otherwise discarded.

### 2.3. Experimental Data Collection

To test AECuration in a large-scale dataset without ground-truth information, we performed *in vivo* experiments on wild-type C57Bl6 mice. All procedures on animals were approved by the Institutional Animal Care and Use Committee of Carnegie Mellon University, and protocols were performed in accordance with the Guide for the Care and Use of Laboratory Animals published by the National Institution of Health. Animals were maintained on a 12 h light-dark cycle with free access to food and water. Four-to-six-week old mice were used for recording neuronal activity following implantation of in-house fabricated probes [68]. Briefly, mice were anesthetized with isoflurane (3% induction, 2% maintenance) and placed on a feedback-controlled heating blanket maintained at 36 ^∘^C (Kent Scientific, Torrington, CT) mounted on a stereotaxic frame (Neurostar, Tubingen-Germany). An incision was made in the skin, and craniotomy was performed by drilling a 1 mm diameter hole above the right hemisphere with the coordinates AP −2 mm and ML +1.5 mm targeting the hippocampus region. A screw hole was made near the lambda region to provide a reference ground to the recording probe. Following craniotomy, a 16-channel in-house probe was inserted at the target site at a depth of DV 1.5 mm to target CA1 regions of the hippocampus. Following insertion, 10 min of recovery was allowed for the acclimatization of tissue to external material before performings pontaneous recordings. Twenty minutes of spontaneous recording, excluding a 30 s initialization period, with a 30 kHz sampling rate across 16 separate channels, was utilized for analysis.

## 3. Results

We tested AEcuration on multiple simulated and experimental electrophysiology datasets. We used a combination of publicly available ground-truth datasets and a large-scale in-house extracellular electrophysiology dataset to perform a broad evaluation. The details of these datasets are shown in table 1. We demonstrated the efficacy of AECuration for preprocessing on the synthetic dataset to evaluate the performance of spike classification with the known ground-truth of each individual spike. For the experimental datasets with ground truth, we demonstrate postprocessing using AECuration with different common spike sorting algorithms, showing that it can be utilized as a universal add-on for diverse experimental conditions to improve performance. For the in-house dataset, which represents typical electrophysiology data recording and lacks a ground-truth, we applied AECuration both for preprocessing and postprocessing to demonstrate that the improvements are significant for real data.

**Table 1.** Details of the datasets. The MEArec datasets are synthetic datasets generated by the MEArec simulator [56]. Publicly available ground-truth datasets with diverse conditions are used to assess AECuration’s ability to generalize and adapt to different experimental environments. The larger in-house dataset, lacking ground-truth information, is used to demonstrate a realistic application scenario.

Dataset	Type	Ground truth	Channels	Species	Regions
MEArec(tetrode)	Synthetic	Simulator	4	Rats	Cortex
MEArec(MEA)	Synthetic	Simulator	100	Rats	Cortex
CRCNS hc1	Experimental	Intracellular electrode	4	Rats	Hippocampal
Kampff	Experimental	Juxtacellular	32	Rats	Cortex
Boyden	Experimental	Patch clamp	32	Mice	Cortex
MEA64C	Experimental	Patch clamp	64	Mice	Retinae
In house	Experimental	N/A	16	Mice	Hippocampal

### 3.1. MEArec dataset

**Figure 6.**
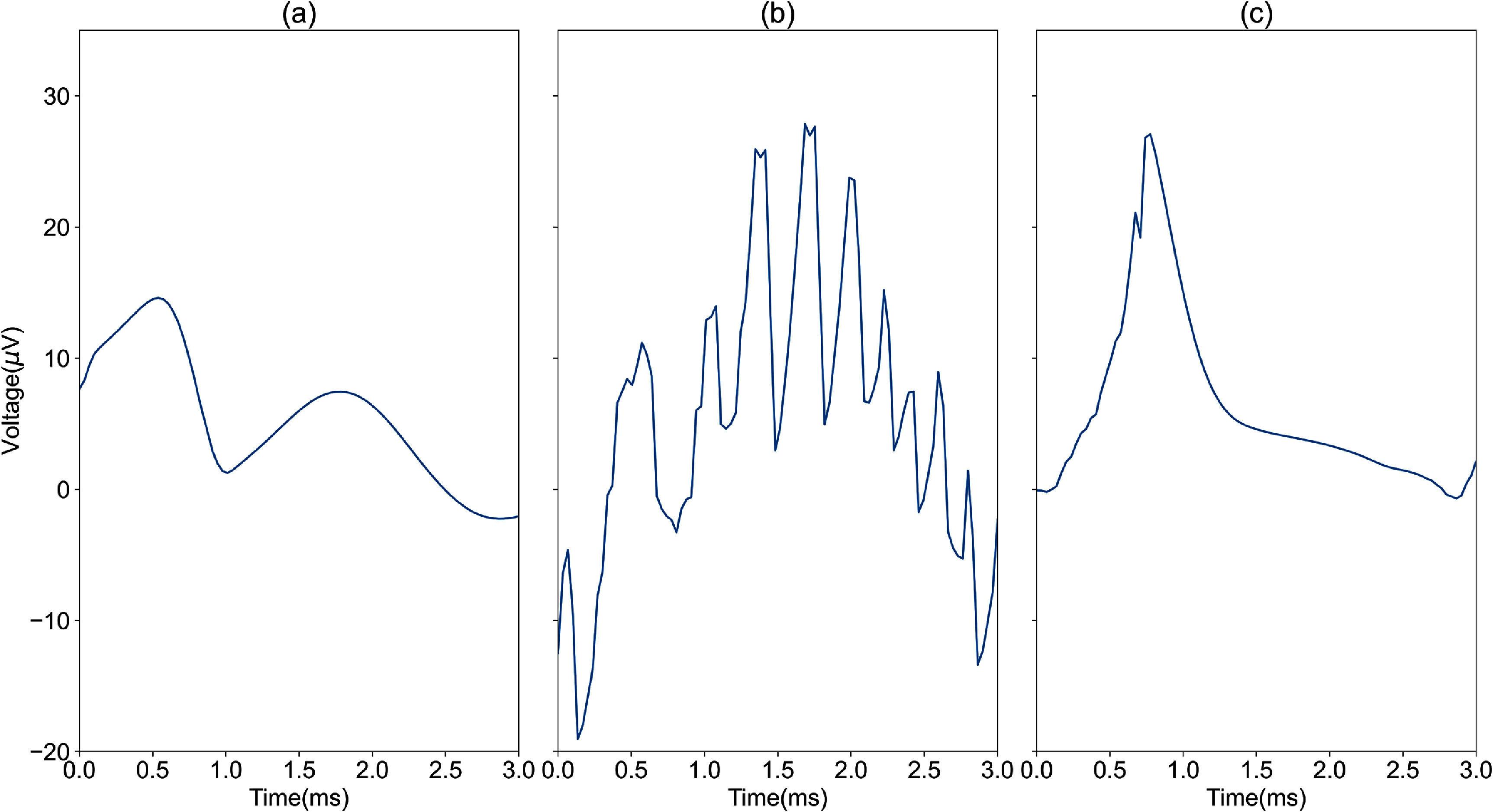
Example spike waveforms representing three groups of spikes in the MEArec dataset. (a) Example of simulated neural spikes from the MEArec simulator. The validation data is generated using the same simulator with different parameters as the training data to ensure these waveforms do not appear in the training dataset. (b) Type I non-neural events have different sources from neural spikes. This is generated using periodic signals with added Gaussian noise to represent heterogeneous signals, like interference and artifacts. (c) Type II non-neural events represent detected events with SNR less than 10. This is generated using the MEArec simulator with a much higher noise level.

To establish the efficacy and accuracy of the AECuration method, we first used simulated data, for which we have the ground truth. The simulated data was generated by the same MEArec simulator used to train the autoencoder but with cell types and orientations not present in the training data to provide a similar context but not the same conditions as training. The MEArec dataset contains both neural spikes and non-neural events. Type I non-neural events were generated using heterogeneous signals, such as sinusoidal, sawtooth, and square waves. Type II non-neural events were generated using the MEArec simulator with a 30 *µ*V Gaussian noise level to represent their remote sources. These types of events are shown in figure 6.

**Figure 7.**
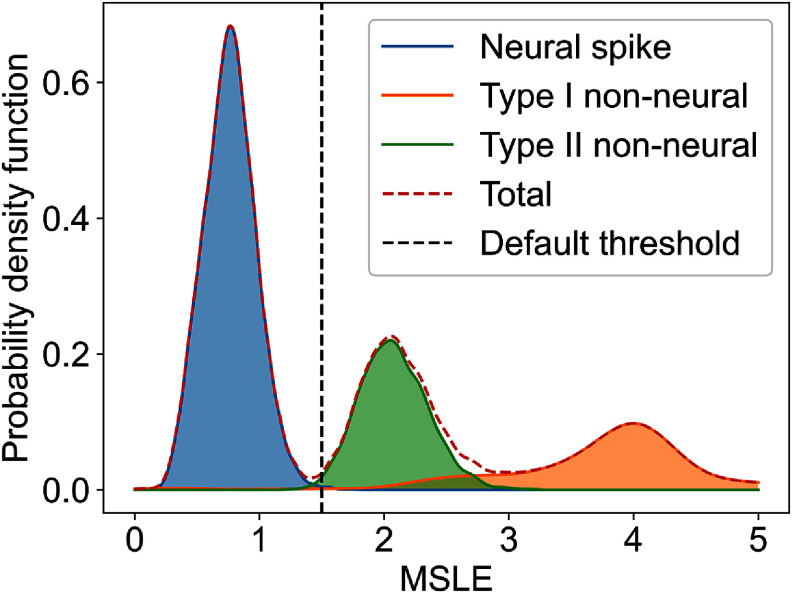
Spike classification on the MEArec dataset, showing probability density function (PDF) for neural spikes and non-neural events (Type I and Type II). The threshold is derived from the training dataset and is set to 1.5 in our implementation. AECuration achieves a classification accuracy of 97.46%, demonstrating its ability to perform pre-clustering waveform filtering.

We validate the preprocessing step on the MEArec dataset by classifying neural and non-neural events, which are shown in figure 7. There are three distinct MSLE distributions that belong to neural spikes and the two types of non-neural events. The neural spikes are concentrated at the low end of the MSLE range, while non-neural events span a broader range. This allows an MLSE threshold of 1.5 to classify neural and non-neural events with >97% accuracy. After the evaluation on the synthetic dataset, we shift our focus to evaluating how AECuration performs as a postprocessing step on experimental ground-truth datasets.

### 3.2. Ground-truth datasets

We show that our method, AECuration, can benefit the existing spike sorting pipelines as a postprocessing method without changing any other step of the spike sorting pipeline, which makes it easy to be integrated into existing workflows. With the help of AECuration, spike sorting performance can be significantly improved by removing non-neural events after clustering. We validate AECuration as a postprocessing step for different spike sorting algorithms on multiple patch-clamp recorded ground-truth datasets from spikeforest [2, 60–63].

Following the same definition in spikeforest, if an event in a sorted unit corresponds to a ground-truth event, with a time stamp difference not exceeding one millisecond, we call it a matched event. For each ground-truth unit, only the sorted unit with the highest matching rate is considered. Here, according to our definition, we consider the matched events as neural spikes and all other events as non-neural events. Since AECuration can selectively remove non-neural events, the postprocessing can increase the sensitivity of spike sorting, which is defined as

\begin{equation*} \textrm{Sensitivity} = \frac{n_\textrm{neural}}{n_\textrm{neural}+n_\textrm{non-neural}},\end{equation*}

where $n_\text{neural}$ is the number of neural spikes events, $n_\textrm{non-neural}$ is the number of non-neural events.

**Figure 8.**
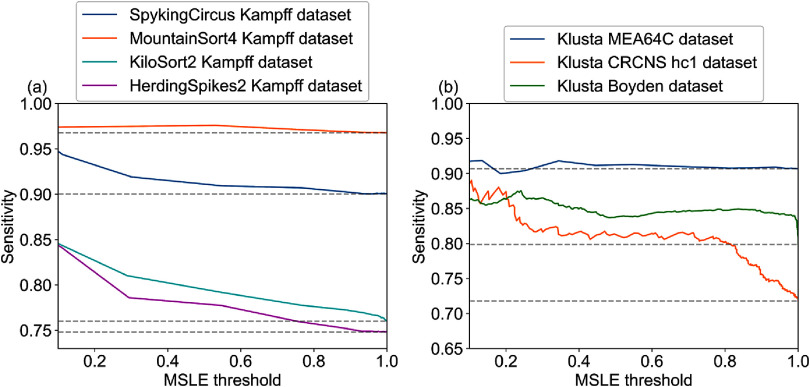
AECuration is applied as a preprocessing step after different spike-sorting algorithms across multiple ground-truth datasets [9, 17, 64–67]. The mean squared logarithmic error (MSLE) threshold is adjusted to show the effect of increasingly selective curation, where an MSLE threshold of 1 corresponds to no additional AECuration filtering. The baseline sensitivity of each spike sorting pipeline with no additional AECuration filtering is indicated by a horizontal dashed grey line. (a) Sensitivity of multiple spike-sorting algorithms using the same recording dataset and AECuration post-processing with varying MLSE threshold. Different spike-sorting algorithms experience varying sensitivity improvements with AECuration. Mountainsort4 consistently maintains the highest sensitivity. (b) Improvement of the Klusta spike-sorting pipeline sensitivity across multiple datasets. The stable and high sensitivity on the MEA64C across AECuration filtering thresholds suggests it contains fewer non-neural events.

AECuration can improve spike sorting without modifying other parts of the spike sorting pipeline, as explained in figure 5. In this configuration, the effect of selectively removing non-neural events is shown by the increasing of sensitivity (figure 8). Adding AECuration selectively removes non-neural events across different spike sorting pipelines to improve the sensitivity, which indicates the quality of spike sorting. The degree of improvement depends on the choice of spike sorting pipeline and quality of recording. However, the same model is applicable in all cases without changing any settings, suggesting a high level of generalizability and that AECuration can be used as an out-of-the-box tool for spike analysis after training in a simulation environment.

Another scalability test is performed to validate AECuration in real-time applications. The computation time of each 3 ms time segment, consisting of loading from memory, autoencoder classification, and comparing all the channels, is calculated for each ground-truth dataset and synthetic dataset with a large number of channels, as shown in table 2. Each computation time is calculated as the average of 1000 operations, excluding the first 10 operations to eliminate the impact from initialization.

**Table 2.** Average per-event computation time of AECuration on different datasets, including the time required for memory loading, autoencoder classification, and channel comparison. Initially the computation time remains fairly constant when increasing the number of channels. However, after the memory I/O reaches its capacity, the computation time linearly increases, indicating a bottleneck in memory rather than computation.

Dataset	Channels	Computation time (ms)
CRCNS hc1	4	28.7
Kampff	32	27.7
Boyden	32	27.9
MEA64C	64	30.1
Synthetic dataset	256	33.8
Synthetic dataset	2048	57.0
Synthetic dataset	8192	148.1
Synthetic dataset	16384	260.8
Synthetic dataset	65536	1050

After showing that AECuration as a postprocessing step can improve the performance of different spike sorting algorithms on the selected ground-truth datasets, we applied it to a larger in-house dataset, which does not have ground-truth information, to show the application of AECuration in a real-world scenario as both a preprocessing and a postprocessing step.

### 3.3. In-house dataset

#### 3.3.1. AECuration preprocessing

First, we apply AECuration as a preprocessing step to the in-house dataset. To evaluate the effect of filtering detected events based on MSLE from AECuration before clustering, we compare multiple quality metrics for the clustered units with and without AECuration preprocessing. We utilize this indirect measurement of sorting quality since without simultaneous patch clamp recording, we do not have access to the ground-truth to determine which events correspond to real neural firings. It is important to know how AECuration performs in this case, since, in most cases, experimental electrophysiology recordings lack ground-truth. We use interpsike interval (ISI) violation to evaluate the clustered units. ISI is the time between consecutive spikes for a unit, which should be larger than a lower bound of the neuronal refractory period according to biophysical constraints. The ISI violation rate is the proportion of consecutive spikes that occur unrealistically close together, violating the refractory period’s biological limit [29]. The ISI violation rate can be used to evaluate the sorting accuracy since a clustered unit with a high ISI violation rate is less likely to originate from neural processes in the brain [20]. We consider an ISI lower than 1.5 ms a violation and a clustered unit with an ISI violation rate lower than 5% an accepted unit. The ISI violation rate can be larger than 100%, if several events are detected within 1.5 ms window and each one is counted as violation. Three groups are used for comparison in this study. The *original* group refers to unprocessed data. All the detected events are used for clustering. The *filter* group refers to the events after AECuration filtering is applied. Only neural spikes are used for clustering. The *random* group is a control group that randomly removes detected events to have the same size as the filter group.

**Figure 9.**
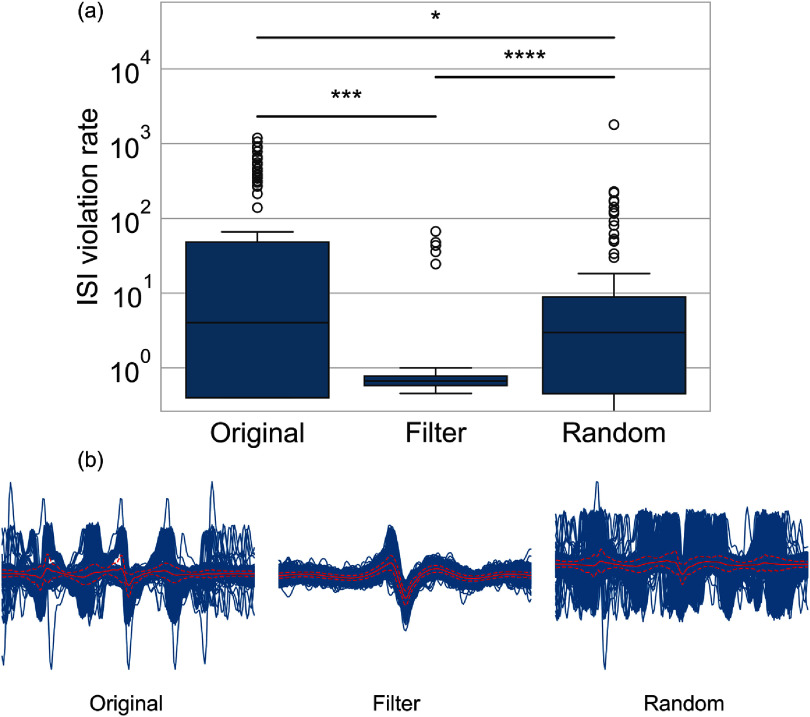
(a) AECuration as a preprocessing step can reduce the ISI violation rates of clustered units. The in-house dataset used the same MSLE threshold as the one used for MEArec dataset, which is 1.5, resulting in removing 25.5% of spikes from clustered units. In the random group, the same proportion of spikes were randomly removed before clustering. (b) Waveforms of the same unit from the original, filter and random groups. The filter group removed most of the non-neural events and has a better template than the original and random groups. This demonstrates the improvement of clustering using AECuration.

The results of this comparison are shown in figure 9 and table 3. The preprocessing can remove non-neural events to decrease the ISI violation rates, which indicates an improvement in quality of clustering.

**Table 3.** Result of preprocessing using AECuration on our in-house dataset. Clustered units refers to the number of units detected by the clustering. Clustered units with low ISI violation rates refer to the number of units with ISI violation rates lower than 5 %. AECuration can reduce the total number of clustered units by removing the non-neural events. Reducing the number of events to cluster also makes the clustering less computationally intensive.

In-house dataset preprocessing			
	Original	Filter	Random
Clustered units	103	69	80
Clustered units with low ISI violation rate	57	59	50
Ratio	55.3%	85.5%	62.5%

There are two advantages to this kind of preprocessing. First, there are fewer data points in clustering to be processed, which can improve the efficiency of clustering. An autoencoder inference combined with MSLE calculation is a much easier computational operation compared to clustering. Second, the clustering is more accurate since the interference from non-neural events is minimized. After demonstrating the efficacy of AECuration for preprocessing to improve clustering, we show how AECuration can also be used as a postprocessing step to replace manual curation.

#### 3.3.2 AECuration postprocessing

The utility of AECuration for postprocessing must also be evaluated using indirect quality metrics due to the lack of ground-truth information for the recording. In addition to the ISI violation rate that we discussed in the previous section, we use the silhouette score to evaluate clustering quality. The silhouette score considers the mean intra-cluster distance *a*_*i*_, the distance between one data point and all the other data points in the same cluster, and the mean nearest-cluster distance *b*_*i*_, the distance between one data point and all the other points in the next closet cluster, for each data point in the whole recording. The silhouette score *S*_*i*_ of each data point *i* is calculated as

\begin{equation*} S_{i} = \frac{b_{i}-a_{i}}{\textrm{max}\left(a_{i},b_{i}\right)}.\end{equation*}

For each data point, *S*_*i*_ ranges from −1 to 1, and a higher value means a better quality of clustering [35]. While ISI violation validates the sorting based on known physiological properties of neurons, the mean silhouette score *S* evaluates the clustering properties. A neural unit has a lower ISI violation rate and higher silhouette score than a non-neural unit. In contrast, hybrid units have scores between neural and non-neural units.

We identified 103 clustered units from the in-house dataset using Mountainsort [17]. AECuration classifies 53 neural units, 39 hybrid units, and 11 non-neural units from the clustered units.

**Figure 10.**
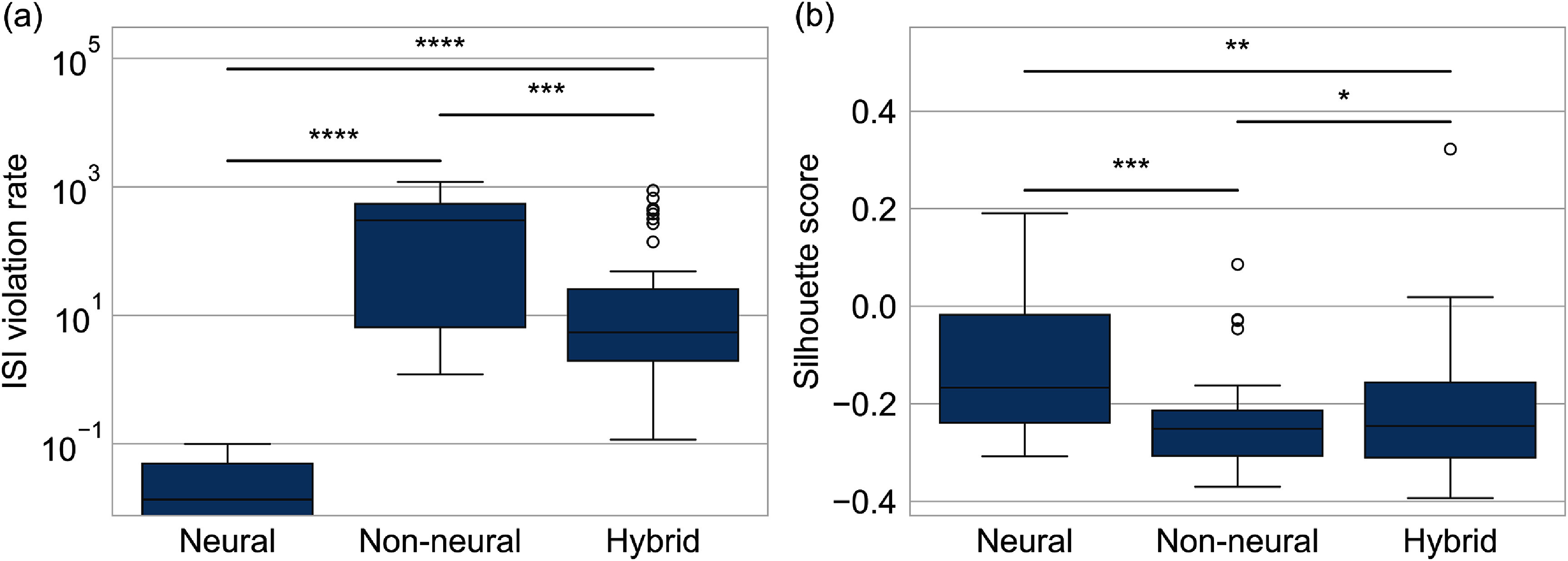
The result of the postprocessing for unit classification on the in-house dataset. (a) ISI violation rates for different types of units. The mean ISI violation rate of each unit indicates whether it is biologically meaningful. (b) Silhouette score for each type of unit. The mean of the silhouette score represents the clustering quality for each unit.

We calculate each unit’s ISI violation rate and silhouette score to validate the classification as shown in table 4 and figure 10.

**Table 4.** Statistics of the unit classification on the in-house dataset.

In-house dataset postprocessing statistics			
	Neural	Non-neural	Hybrid
ISI violation rate(%)	0.02	340.83	83.16
Silhouette score	−0.12	−0.23	−0.22

These results show that neural units, as identified by AECuration, have better quality than hybrid and non-neural units, both physiologically and statistically.

**Figure 11.**
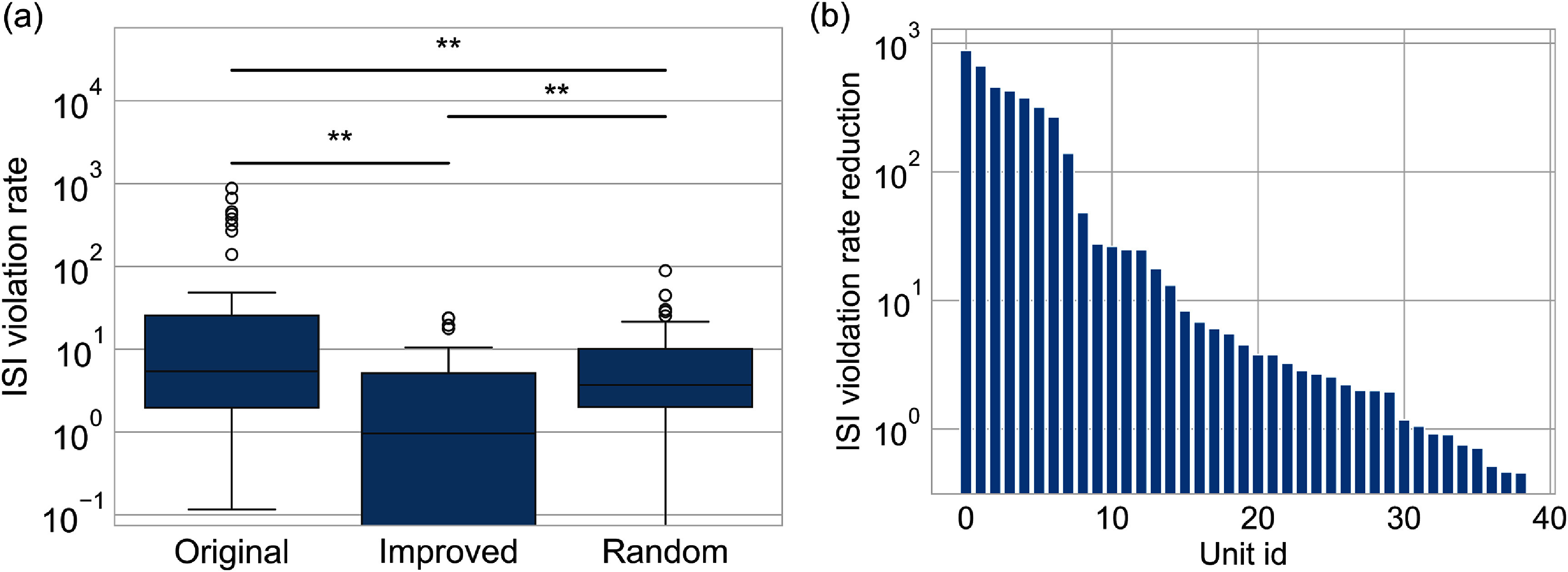
The result of AECuration postprocessing for filtering hybrid units from the in-house dataset. The original group refers to the hybrid units without filtering using AECuration. The improved group refers to the hybrid units after filtering AECuration. The random group refers to the control units for which the same number of spikes are randomly removed from the original group. (a) ISI violation rates for each group. (b) Improvement of ISI violation rate for each hybrid unit in the improved group after filtering.

Typical manual curation will discard both non-neural and hybrid units. In contrast, AECuration can further process hybrid units and utilize their information. AECuration can discard non-neural events to refine hybrid units. After removing non-neural events based on an MLSE threshold of 1.5, the refined unit should have a lower ISI violation rate than the original unit. The silhouette score is not used here; the silhouette score may not reflect the improvement in a specific unit but in other units, since the calculation of the silhouette score involves a comparison between all the units [69]. Three groups are compared. The original group contains the unfiltered hybrid units. The improved group is the refined group with non-neural events removed, and the random group is a control group with spikes randomly removed. The result is shown in figure 11.

**Figure 12.**
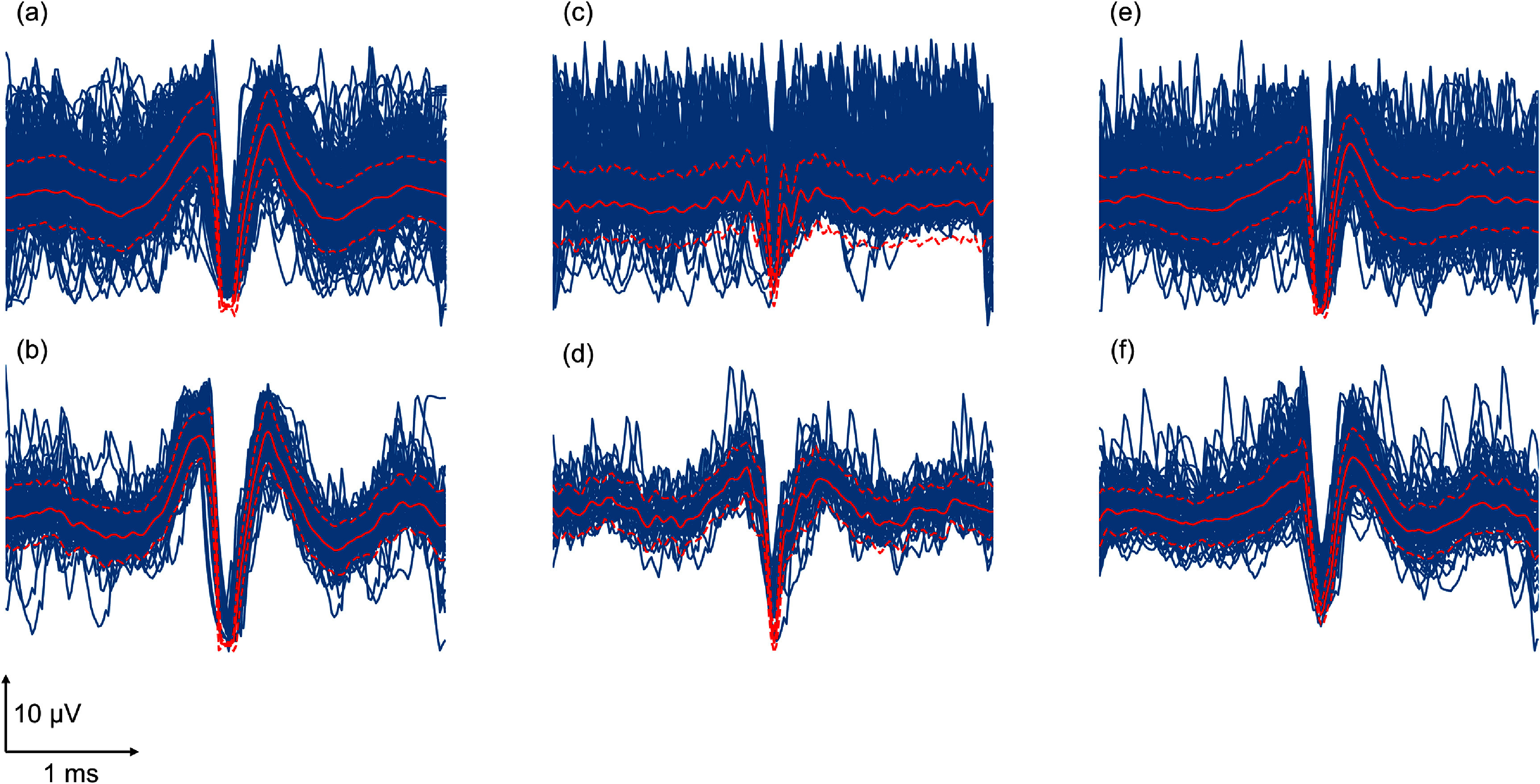
Examples of unit waveforms before and after removing non-neural events from hybrid units using AECuration. The first row (a)(c)(e) shows the original waveforms and the second row (b)(d)(f) shows the filtered waveforms. Blue waveforms show individual events. Solid red waveforms show the template for the unit. Dashed red curves show one standard deviation of the unit. Standard deviation decreases from 80.24 to 43.11 for filtered waveforms.

Selectively removing non-neural events can decrease the ISI violation rate compared to the original group and random group. This shows that removing a subset of events according to MSLE achieves better unit quality. As shown in the following examples in figure 12, the improved quality of clustering can also be observed via reduction of variance in the unit waveform.

## 4. Discussion

While different automatic spike sorting frameworks have been developed, automated assessment of sorted units has remained elusive. Here, we demonstrated a new automatic curation algorithm, called AECuration that analyzes the neural data spike-by-spike. Furthermore, we demonstrate the potential of applying the autoencoder to spike sorting in both preprocessing and postprocessing configurations. AECuration is highly generalizable from a synthetic dataset to experimental recordings. Finally, our method can be readily integrated into most of the existing spike sorting pipelines as an add-on.

AECuration uses a new time domain approach to automate curation. Previous automatic curation methods based on statistical analysis overlooked the time-domain features of individual spikes [20]. In contrast, AECuration can process individual events based on features of the waveform, which contains biologically meaningful patterns.

A challenge for spike sorting assessment is the lack of ground truth in most experimental electrophysiology recordings. To address this limitation, our autoencoder is trained on synthetic data, where a reliable ground truth is available, and then applied to experimental recordings. Our synthetic training dataset rigorously models neuronal biophysical properties [56]. The use of synthetic data is a well-established practice and has been employed in diverse domains such as robotics, computer vision, and neuroscience to provide a controlled experimental environment [70–72]. A key advantage to using synthetic recordings is that the dataset can be made arbitrarily large and varied, with a diversity of cell types, orientations, and recording configurations, to increase the generalizability of the resulting autoencoder model.

A novel capability of our method is to discriminate and extract neural spikes from a noise-contaminated unit, which would typically be discarded during manual curation. AECuration can be used to remove individual non-neural events from a detected unit to improve it, which cannot be achieved without the assessment of individual spikes. The extracted subsets perform better and can be used in the following analyses, expanding available recordings for neuroscience research.

The scalability of AECuration is critical for real-world applications in high-density electrophysiology, especially for applications like brain-machine interfaces, AECuration must meet strict time constraints. The current processing time of 28 ms per event, as we have discussed in table 4, is good enough for offline analysis and can be effectively scaled using hardware parallelization since each event classification is independent of the others. Hardware acceleration using devices like field programmable gate arrays (FPGAs) can accelerate algorithms with parallelization. These hardware platforms are designed for specific tasks to improve efficiency and power consumption and have been successfully used to accelerate custom spike sorting pipelines [73, 74]. We could also optimize memory usage by optimizing the quantization of the model.

This work demonstrates the ability to use the temporal properties of individual spikes to assess and improve the spike sorting process. Our AECuration method discussed in this paper is a significant advance in automating the assessment of sorted spikes, improving both the efficiency and accuracy of spike sorting methods and eliminating human error.

## Data Availability

The data that support the findings of this study are openly available at the following URL/DOI: https://github.com/az123zx123/AECuration.
